# Hybrid Stem Cell States: Insights Into the Relationship Between Mammary Development and Breast Cancer Using Single-Cell Transcriptomics

**DOI:** 10.3389/fcell.2020.00288

**Published:** 2020-05-08

**Authors:** Tasha Thong, Yutong Wang, Michael D. Brooks, Christopher T. Lee, Clayton Scott, Laura Balzano, Max S. Wicha, Justin A. Colacino

**Affiliations:** ^1^Department of Environmental Health Sciences, School of Public Health, University of Michigan, Ann Arbor, MI, United States; ^2^Department of Electrical Engineering and Computer Science, University of Michigan, Ann Arbor, MI, United States; ^3^Department of Internal Medicine, Michigan Medicine, University of Michigan, Ann Arbor, MI, United States; ^4^Department of Biostatistics, School of Public Health, University of Michigan, Ann Arbor, MI, United States; ^5^Center for Computational Medicine and Bioinformatics, University of Michigan, Ann Arbor, MI, United States; ^6^Department of Nutritional Sciences, School of Public Health, University of Michigan, Ann Arbor, MI, United States

**Keywords:** stem cells, breast cancer, single-cell RNA sequencing, hybrid, epithelial, mesenchymal, pregnancy

## Abstract

Similarities between stem cells and cancer cells have implicated mammary stem cells in breast carcinogenesis. Recent evidence suggests that normal breast stem cells exist in multiple phenotypic states: epithelial, mesenchymal, and hybrid epithelial/mesenchymal (E/M). Hybrid E/M cells in particular have been implicated in breast cancer metastasis and poor prognosis. Mounting evidence also suggests that stem cell phenotypes change throughout the life course, for example, through embryonic development and pregnancy. The goal of this study was to use single cell RNA-sequencing to quantify cell state distributions of the normal mammary (NM) gland throughout developmental stages and when perturbed into a stem-like state *in vitro* using conditional reprogramming (CR). Using machine learning based dataset alignment, we integrate multiple mammary gland single cell RNA-seq datasets from human and mouse, along with bulk RNA-seq data from breast tumors in the Cancer Genome Atlas (TCGA), to interrogate hybrid stem cell states in the normal mammary gland and cancer. CR of human mammary cells induces an expanded stem cell state, characterized by increased expression of embryonic stem cell associated genes. Alignment to a mouse single-cell transcriptome atlas spanning mammary gland development from *in utero* to adulthood revealed that NM cells align to adult mouse cells and CR cells align across the pseudotime trajectory with a stem-like population aligning to the embryonic mouse cells. Three hybrid populations emerge after CR that are rare in NM: *KRT18+*/*KRT14+* (hybrid luminal/basal), *EPCAM+/VIM+* (hybrid E/M), and a quadruple positive population, expressing all four markers. Pseudotime analysis and alignment to the mouse developmental trajectory revealed that E/M hybrids are the most developmentally immature. Analyses of single cell mouse mammary RNA-seq throughout pregnancy show that during gestation, there is an enrichment of hybrid E/M cells, suggesting that these cells play an important role in mammary morphogenesis during lactation. Finally, pseudotime analysis and alignment of TCGA breast cancer expression data revealed that breast cancer subtypes express distinct developmental signatures, with basal tumors representing the most “developmentally immature” phenotype. These results highlight phenotypic plasticity of normal mammary stem cells and provide insight into the relationship between hybrid cell populations, stemness, and cancer.

## Introduction

As the field of cancer biology has evolved, a growing body of work has reinforced the critical role of stem-like cells in cancer. Due to long-observed similarities between embryonic development and oncogenesis, cancer is often considered a disease of “dysregulated development” (Ma et al., 2010; Reya et al., 2001). A characteristic shared by stem cells and cancer cells is cellular plasticity – the ability to transition and adopt alternative cell fates in response to environmental signals and stressors (Thong et al., 2019). Plasticity is crucial for stem cells during embryonic development, for example during gastrulation when epiblast cells undergo the epithelial to mesenchymal transition (EMT) to form mesoderm which gives rise to the mesenchyme (Kalluri and Weinberg, 2009). In adult stem cells, plasticity plays an important role in homeostasis and wound repair. This is demonstrated by adult tissue stem cells in the liver and intestinal epithelium which have been shown to de-differentiate or even *trans-*differentiate into cell types of a different lineage in order to replace damaged cells (Merrell and Stanger, 2016). For cancer cells in tumors of epithelial origin, EMT plasticity and its reverse MET, are crucial for primary tumors to be able to adopt mesenchymal characteristics in order to disseminate, metastasize, and re-epithelialize at the metastatic site (Tam and Weinberg, 2013).

Emerging evidence now suggests that these transitions occur along a continuum rather than as discrete switches in cell state. Transitioning hybrid cells exhibiting phenotypic markers of multiple cell states (epithelial/mesenchymal and luminal/basal) have been identified by us and others in both normal and carcinogenic breast tissue (Grosse-Wilde et al., 2015; Jolly et al., 2015; Colacino et al., 2018). These cellular states are defined by the co-expression of known marker genes, for example the epithelial marker *EPCAM* and the mesenchymal marker *VIM*, or the luminal marker *KRT18* and the basal/myoepithelial marker *KRT14*. Additionally, recent evidence shows that these hybrid cells exist in metastable states, not just as transient hybrids (Jolly et al., 2016). These hybrid populations are of particular interest due to their implicated role in promotion of tumorigenesis, metastasis, and aggressiveness of breast cancer (Jolly et al., 2015). There are a limited number of studies which have observed these hybrid populations in the normal breast, and of these, the low proportions of hybrid cells identified have made them challenging to characterize.

In this study, we integrate multiple single-cell RNA sequencing datasets from the human and mouse in order to characterize the cell state distributions of the normal mammary (NM) gland throughout the life course, as well as after being perturbed into an enriched stem cell state following *in vitro* culture using the conditional reprogramming (CR) method (Liu et al., 2017). Through a combined analysis of single cell RNA-seq data with bulk human breast cancer transcriptomics from the Cancer Genome Atlas (TCGA), we investigate mammary stem cell populations and hybrid cell states, elucidating roles for these cells in mammary gland development and cancer.

## Materials and Methods

### Human Tissue Procurement

Tissue procurement was approved by the University of Michigan Institutional Review Board (HUM00042409). Normal mammary (NM) tissue was obtained from voluntary reduction mammoplasties performed at the University of Michigan hospital. Samples were processed following the protocol of Dontu et al. (2003) by enzymatic and mechanical digestion into single cell suspensions, as previously described (Colacino et al., 2016).

### Conditional Reprogramming

NM cells isolated from mammoplasty dissociation were co-cultured with irradiated 3T3 J2 mouse fibroblasts (Kerafast) using F-media in adherent conditions according to the protocol of Liu et al. (2012, 2017). To establish an effective feeder layer, irradiated J2 fibroblasts were plated at a density of 12,000 cells per cm (Ma et al., 2010; Liu et al., 2017). Once plated, the co-cultured cells were incubated in a humidified incubator at 37°C/5% CO_2_. Conditionally reprogrammed (CR) cells were allowed to grow up to 80% confluence and 0.05% trypsin/EDTA (Gibco, cat. no. 25300054) was used to differentially trypsinize cells from the adherent culture dishes. Differential trypsinization detaches irradiated J2s first, leaving behind an enriched population of CR mammary cells to be used for experimentation or cryopreservation.

To culture and irradiate J2 fibroblasts, cells were plated with J2 media in T-150 flasks (250,000–500,000 cells) and allowed to grow up to 80% confluence. J2s were carefully cultured to not exceed 90% confluence. Once confluent, J2s were trypsinized using 0.05% trypsin/EDTA, resuspended in J2 media, and placed on ice to be transported for irradiation. J2s were irradiated at 30 grays for 6 min, viability was assessed via acridine orange/propoidium iodide staining. Irradiated J2 cells were cryopreserved at 300,000–500,000 cells per vial in recovery cell culture freezing medium (Gibco, cat. no. 12648010). Following irradiation, 100,000 irradiated J2s and non-irradiated controls were plated for comparison in order to ensure success of irradiation in halting cell proliferation.

J2 media was prepared by combining 500 mL DMEM (Gibco, cat. no. 11965-092), 50 mL bovine calf serum (ATCC cat. no. 30-2030), 5.5 mL 200 mM L-glutamine (Gibco, cat. no. 25030081), and 5.5 mL 100X Pen-Strep (Gibco, cat. no. 15140122). F-media was made by combining 623.83 μL of 12 mM Rho-associated kinase (ROCK) inhibitor Y-27632 (Cayman Chemical, cat. no. 10005583), 194.48 μL of 96 μg/mL hydrocortisone (STEMCELL Technologies, cat. no. 07925), 8.98 μL of 10 μg/mL epidermal growth factor (STEMCELL Technologies, cat. no. 78006.1), 935 μL of 4 mg/mL insulin (Invitrogen-LifeTechnologies, cat. no. 12585014), and 62.83 μL of 1.2 μM cholera toxin (Sigma-Aldrich, cat. no. C8052) and 561 mL of complete DMEM (500 mL DMEM (Gibco, cat. no. 11965-092), 50 mL heat inactivated fetal bovine serum (Sigma-Aldrich cat. No F4135), 5.5 mL 200 mM L-glutamine (Gibco, cat. no. 25030081), and 5.5 mL 100X Pen-Strep (Gibco, cat. no. 15140122).

### Single Cell RNA-Sequencing

NM cells and their CR counterparts (*n* = 3 pairs) were removed from liquid nitrogen storage and individually thawed, centrifuged, and counted. Cell mixtures were diluted with 0.01%FBS+PBS solution to achieve a final concentration of 100 cells/uL for each 5 mL sample (500,000 cells/sample). Samples were placed on ice and processed for drop-seq analysis according to the protocol of Macosko et al. (2015).

The drop-seq microfluidic device was assembled and calibrated to dispense oil droplets (Bio-Rad cat # 186-4006), cells, and Barcoded Bead SeqB (Chemgenes) beads at optimal velocity. Samples were loaded into the apparatus and the cell and microbead containing droplets were collected in 50 mL conical tubes (Falcon). Following droplet collection, a series of wash, transfer, and centrifuge steps were performed in order to prepare the microbeads for sequencing.

After bead purification the following workflow was performed in order to generate DNA for sequencing. To generate cDNA strands from RNA hybridized to bead primers, RT mix was added to the microbeads and incubated. Following incubation, microbeads were rinsed and resuspended in exonuclease mix to remove excess bead primers that did not capture any RNA, rinsed, and then prepped for PCR. A 13 cycle PCR program was run to amplify cDNA and the generated cDNA library was then purified and analyzed on a BioAnalyzer High Sensitivity Chip. The purified cDNA was then tagmented using Nextera XT, PCR amplified, analyzed again using the BioAnalyzer. Following these steps, the library was sequenced on a NextSeq 500 (Illumina) at the University of Michigan Advanced Genomics Core Facility.

### Single-Cell Data Analyses

#### Raw Data Processing

The “Drop-seqAlignmentCookbookv1.2Jan2016” software was used to transform raw sequencing data into gene expression measurements for each individual cell. The paired end reads were aligned to a mixed human (hg19) and mouse (mm10) reference genome and then grouped by cell according to the cell bar code. Next, a digital expression matrix was generated from the unique molecular identifier (UMI) counts for each gene in each cell. We performed quality control (QC) filtering on the raw data, filtering out cells with greater than 5% of mitochondrial genes and fewer than 200 total genes. Following QC filtration, we performed global log-normalization, scaling by percent mitochondrial genes, detection of highly variable genes, and principal component analysis dimension reduction. QC and downstream data processing were performed using the Seurat R package v3 unless specified otherwise (Satija et al., 2015).

#### Unbiased Clustering and Cell Type Identification

Graph based unbiased clustering and PCA based tSNE dimension reduction were performed on NM, CR, and pooled NM and CR samples at a resolution of 0.5. Cell type identification was performed by identifying marker genes for individual clusters (Supplementary Tables S1, S2) as well as assessing expression of a pre-selected a panel of cell type marker genes. Cluster markers were identified using the FindMarkers function in Seurat and marker gene expression was assessed using the FeaturePlot and VlnPlot functions. Due to the differences in numbers of cells captured and analyzed between the NM and CR samples, we performed a normalized analysis by randomly down-sampling each individual sample to 200 cells and performing the same clustering and marker gene assessment as performed on the full dataset.

#### Differential Gene Expression Analysis

In order to isolate epithelial subsets of NM and CR cells for direct comparison, we filtered out stromal and immune cells from NM samples, and mouse cells were filtered out from CR samples. We performed differential expression analysis between pooled epithelial CR and NM samples with PQLseq, which uses a penalized quasilikelihood and a heredity correlation matrix (Sun et al., 2019). The hereditary matrix was designed with the hierarchical data structure in mind in order to account for random effects of individual samples. Doing so prevents any one individual with a large number of cells relative to any other to dominate the analysis, and provides a more powerful analysis compared to a naïve approach. Differential gene expression between the NM and CR cells within each individual was performed using the FindMarkers function in Seurat. DEGs between NM and CR for each individual were plotted by average log2FC and correlation coefficients were calculated for each comparison.

#### Embryonic Stem Cell Score

To estimate the similarities of the gene expression pattern of each cell to an embryonic stem cell, we calculated an “embryonic stem cell gene expression score.” The proportion of total reads which belong to genes in the Embryonic Stem Cell Core set from the Gene Set Enrichment Analysis (GSEA) were calculated on a per cell basis (Wong et al., 2008). A higher stem cell score can be interpreted as a greater proportion of reads for a given cell being derived from embryonic stem cell-associated genes.

#### Transcription Factor and Enrichment Analyses

The top 1000 DEGs in CR compared to NM by log2FC were uploaded to the Enrichr web server to identify ENCODE and ChEA transcription factors enrichment (Kuleshov et al., 2016). To characterize the enrichment of the mammary stem cell and luminal progenitor gene sets reported by Lim et al. (2009) and ROCK pathway gene signatures, each of these gene sets was overlapped with CR DEGs. The overlapped genes were then plotted by CR vs. NM log2FC to visualize gene signature enrichment.

#### Identification of Hybrid Populations

Hybrid populations were identified in using expression of *KRT14*, *KRT18*, *VIM*, and *EPCAM*. Cells expressing marker genes at the 50th percentile or greater were deemed “high expressors.” High expressors for the *KRT14/KRT18* or *EPCAM/VIM* marker combinations were identified as “double positive” hybrids, and high expressors for all four marker genes were identified as “quadruple positive” hybrids. Differential gene expression analysis between quadruple hybrids and all other NM and CR epithelial cells was performed using FindMarkers in Seurat. Differentially expressed genes upregulated in quadruple hybrid cells were intersected with the MSigDB Hallmark Epithelial Mesenchymal Transition gene set (*n* = 200) in order identify EMT related genes expressed in quadruple hybrids.

### Integration and Alignment of NM, CR, Bach, Nguyen, and the Cancer Genome Atlas (TCGA) Breast Cancer RNA-seq Samples to the Giraddi Mouse Mammary Transcriptome Atlas

#### Dataset Descriptions

To contextualize our findings in NM and CR cells, we also performed an integrated analysis with three other single cell RNA-seq mammary gland datasets generated from mice and humans, as well as a comparative analysis using bulk breast cancer RNA-seq data from TCGA. The “Bach” dataset contains single cell RNA-seq profiling of mouse mammary gland from four developmental stages: nulliparous, mid gestation, lactation, and post involution (Bach et al., 2017). The “Nguyen” dataset is comprising of single cell RNA-seq profiling of human mammary gland generated from adult voluntary reduction mammoplasty patients (Nguyen et al., 2018). The “Giraddi” dataset is comprized of single cell RNA-seq data from multiple timepoints during the lifecourse: embryonic day 16, embryonic day 18, postnatal day 0, postnatal day 4, and adult (Giraddi et al., 2018). Bulk RNA-seq counts of TCGA breast tumors were obtained from the National Cancer Institute’s genomic data commons portal using the TCGAbiolinks R package (Colaprico et al., 2016).

#### Data Pre-processing

The raw counts data of NM, CR, Bach, and Nguyen cells were normalized using either the “multiBatchNorm” or the “normalize” function in the R package scran. For gene filtering, a modified version of the CORGI algorithm was used on the Nguyen and Giraddi datasets, hereinafter referred to as “CORGI genes” (Giraddi et al., 2018; Nguyen et al., 2018). The CORGI gene filtering algorithm works by randomly sampling subsets of genes and scoring the subsets based on the structuredness of the data (Wang et al., 2019a). Genes that lead to more structured data are encouraged and vice versa. The TCGA dataset was pre-processed in the same way as the single-cell samples.

#### Down-Sampling and Cell Selection

To generate the Giraddi reference dataset used for alignment, the full mouse mammary dataset was randomly down-sampled to 1000 cells spanning the four developmental stages (embryonic day 16, embryonic day 18, gestational day 4, and adult). Proportions of cells in the generated reference dataset reflect the proportions of cells from each developmental stage in the original dataset. The Bach mouse dataset was down-sampled by randomly selecting 250 cells from each of the four adult developmental stages (nulliparous, mid-gestation, lactation, and post-involution) for a total of 1000 cells. NM, CR, and Nguyen datasets were also randomly down-sampled to 1000 cells each.

#### Batch Correction

For batch correction, the “mnnCorrect” function in scran was used with default parameters on the logcounts on CORGI genes. The Giraddi dataset was input into the mnnCorrect as the first argument, i.e. as the reference atlas. Subsequently, the NM, CR, Bach, Nguyen, and TCGA samples were then projected onto the Giraddi developmental trajectory for comparative analysis.

#### Pseudotime Analysis

In order to place the various datasets onto a developmental timeline, we leveraged the Giraddi mouse atlas as a reference. Pseudotime is computed directly onto the two-dimensional PCA plots by taking the dot product with an “arrow-of-time” vector that differentiates between the adult and embryonic cell populations in the Giraddi dataset. The same arrow-of-time vector was then applied to the NM, CR, Bach, Nguyen, and TCGA samples. A generalized linear model was used to determine significantly different pseudotime means between TCGA subtypes.

#### Dataset Availability

The drop-seq data for the NM and CR samples are available on the Gene Expression Omnibus (GSE146792).

## Results

### Normal Mammary Cells Contain a Mixture of Stromal and Epithelial Cells and Cluster by Subtype

**FIGURE 1:**
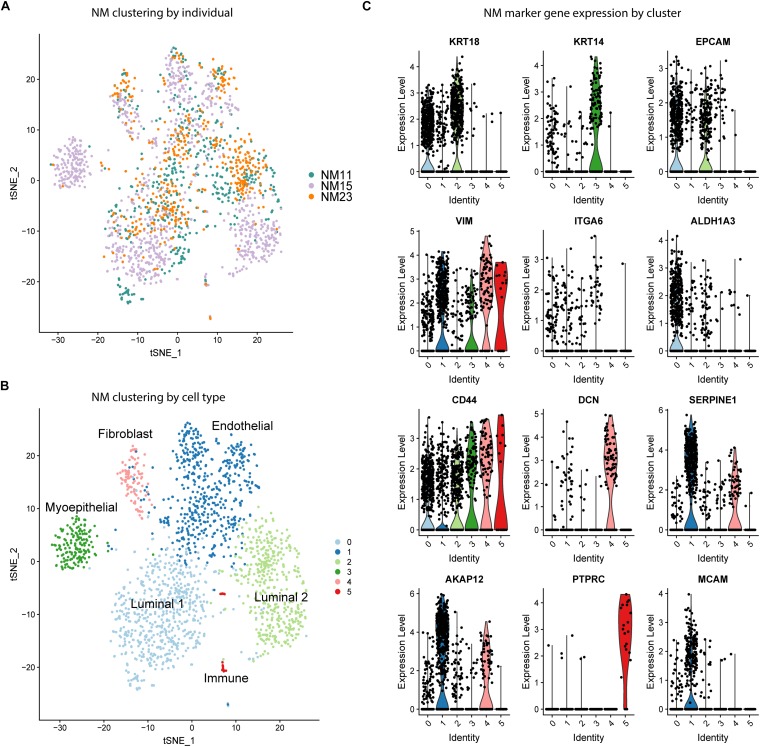
Unbiased clustering and cell type identification of NM cells. **(A)** tSNE dimension reduction of NM samples colored by individual. **(B)** Unbiased clustering of NM samples colored by cell cluster. **(C)** Expression of known cell type marker genes by cluster across all NM samples.

As a first step toward characterizing the distribution of phenotypic states of epithelial cells in the human mammary gland, we performed unbiased clustering of NM scRNA-seq data to determine the cell types and proportions present in the samples. Samples were analyzed from three individuals, here termed “NM11”, “NM15”, and “NM23”. tSNE visualization revealed that the majority of clusters contained cells from each individual (Figure 1A). To determine the identity of the six clusters (Figure 1B), a panel of known breast cell type and stem cell marker genes (Figure 1C) along with the top marker genes for each cluster identified by Seurat (Supplementary Table S1), were used to characterize the clusters. The two major epithelial subtypes of the breast were identified by *KRT18* (luminal) and *KRT14* (myoepithelial) expression (Figure 1C; O’Hare et al., 1991; Abd El-Rehim et al., 2004). Clusters 0 and 2 (Figure 1B) represent two distinct luminal populations which both highly express epithelial marker *EPCAM* but differentially express stem cell marker *ALDH1A3*, which is preferentially expressed in cluster 0 (Trzpis et al., 2007; Marcato et al., 2011). Mammary stem cell markers *ITGA6* and *CD44* also exhibited varying expression by cluster, with *ITGA6* showing low expression in clusters 0-3 and *CD44* exhibiting moderate to high expression across all clusters (Al-Hajj et al., 2003). The myoepithelial Cluster 3 was almost entirely composed of cells from one individual (NM15), indicating variation in cell type proportions by individual. We identified cluster 4 identified as fibroblasts (*DCN*), cluster 1 as endothelial cells (*SERPINE1*, *AKAP12*), and cluster 5 as a small population of immune cells (*PTPRC*). Thus, prior to CR, normal mammary cells are composed of a mixture of stromal, immune, and epithelial cells and cluster primarily by cell type.

#### Conditionally Reprogrammed Mammary Cells Cluster by CR Status and by Individual

**FIGURE 2:**
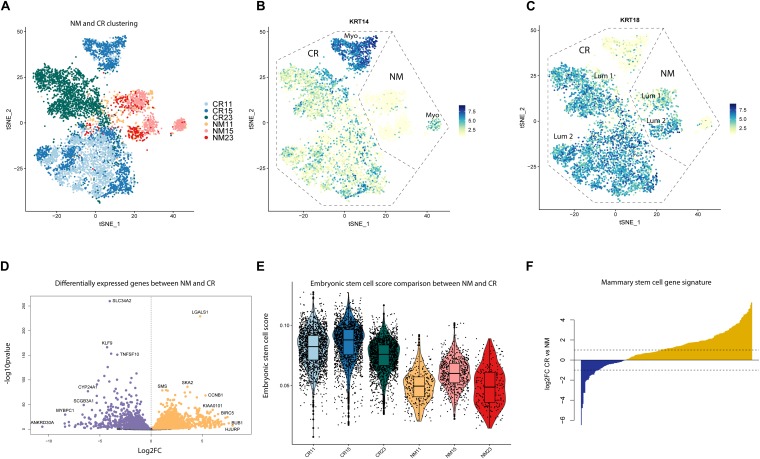
Unbiased clustering and differential gene expression between NM and CR. **(A)** tSNE dimension reduction of NM and CR samples by individual. **(B)** FeaturePlots of myoepithelial marker gene (*KRT14*) and **(C)** luminal marker gene (*KRT18*) expression. **(D)** Differentially expressed genes between NM and CR epithelial cells. Significantly upregulated genes in CR (FDR < 0.05) are colored in orange. Significantly upregulated genes in NM are colored in purple. **(E)** Distribution of cells from NM and CR samples scored by embryonic stem cell gene expression. **(F)** Comparison of overlap between NM and CR differentially expressed genes and the mammary stem cell (MaSC) gene expression signature reported in Lim et al. (2009). Yellow genes indicate MaSC genes more highly expressed in CR vs. NM.

Marker analysis of the CR samples revealed that samples were depleted of fibroblasts, endothelial cells, and immune cells, but retained luminal and myoepithelial populations (Supplementary Figures S1A,B). We identified two clusters (7 and 8) of mouse fibroblasts, using the mouse gene *Gapdh* as a marker, which we excluded in downstream analyses (Supplementary Figure S1B and Supplementary Table S2). To characterize CR alterations specifically in epithelial cells, we grouped NM and CR epithelial cells together for analysis. Unbiased clustering of the NM and CR cells revealed that NM samples remained relatively well mixed amongst each other, whereas CR samples distinctly clustered by individual (Figure 2A). While CR11 and CR15 exhibited some overlap in clustering, CR23 remained distinct from the other samples. Samples clustered by CR status along tSNE_1 and both NM and CR samples clustered as myoepithelial and luminal cells (Figures 2B,C). *KRT14* was selectively expressed in NM and CR myoepithelial populations, however, *KRT18* expressing CR cells also co-expressed moderate levels of *KRT14*. To determine if this clustering behavior was representative of CR gene expression alterations or due to the greater proportion of CR to NM cells, the same clustering and marker gene identification was performed on a randomly down-sampled subset comprised of 200 cells from each NM and CR sample. This subset of cells displayed the same clustering patterns and marker gene expression as the full dataset (Supplementary Figures S1C–F). The co-expression (*KRT18*/*KRT14*) of luminal and myoepithelial markers was the first indication that the CR process could induce a hybrid state phenotype worthy of further investigation.

### Conditionally Reprogrammed Mammary Cells Differentially Express Breast Cancer and Stem Cell Associated Genes

To gain mechanistic insight into the effects of the CR process, we compared gene expression patterns between NM and CR cells with differential gene expression (DGE) analysis. DGE between the NM and CR epithelial cells resulted in 3177 genes differentially expressed between the two cell populations (FDR < 0.05) (Figure 2D; Supplementary Table S3). DGE was also conducted between the NM and CR cells of each individual and the overlap of differentially expressed genes (DEGs) was compared between individuals (Supplementary Figure S2A and Supplementary Table S4). DEGs by individual were consistent with those found in combined NM and CR analysis, with both analyses identifying *LGALSI* as one of the most differentially upregulated genes in CR. Comparing DEGs between NM and CR by individual also revealed that the DEGs between samples 11 and 15 are highly correlated with each other (*r* = 0.896) whereas DEGs between samples 11 and 23 (0.713) and between samples 15 and 23 (0.79) are less well correlated. Because the CR process requires the ROCK pathway small molecule inhibitor Y-27632, we assessed DEGs overlapping with ROCK associated pathway genes (Supplementary Figure S2E and Supplementary Table S5). Unsurprisingly, *ROCK2* was the most significantly downregulated gene in this pathway (log2FC = −1.25) in CR cells. We input the top 1000 upregulated DEGs in CR to the Enrichr web server to identify transcription factors likely driving this process. Gene targets of known stem cell associated transcription factors *E2F4, FOXM1, BRCA1, SOX2, KLF4*, and *MYC* were all identified as enriched in CR upregulated genes (Supplementary Figure S2F and Supplementary Table S6).

To further investigate whether NM and CR cells exhibit differences in expression of stem cell associated genes, we performed analyses using overall gene expression as well as NM and CR DEGs. We estimated how “embryonic stem cell-like” each cell was by calculating the proportion of total transcripts annotated to embryonic stem cell (ESC) associated genes (Supplementary Table S7) expressed in each NM and CR sample (Wong et al., 2008). CR samples had higher ESC scores than their NM counterparts (Figure 2E), providing further evidence that CR cells express a more developmentally immature phenotype. To further characterize this phenotype in comparison to stem and progenitor cells in the normal breast, we overlapped NM and CR DEGs with mammary stem cell (MaSC) and luminal progenitor gene expression signatures reported by Lim et al. (2009) (Supplementary Tables S8, S9) (Lim et al., 2009). Of the MaSC associated DEGs, 211/282 of the genes were upregulated in CR (Figure 2F), whereas only 68/144 luminal progenitor associated DEGs were upregulated in CR (Supplementary Figure S2D). Together, these analyses suggest that the CR process enriches for a stem cell-like state, and that the CR transcriptomic signature resembles ESCs and MaSCs.

### Conditionally Reprogrammed Cells Reflect a More Developmentally Immature Phenotype

**FIGURE 3:**
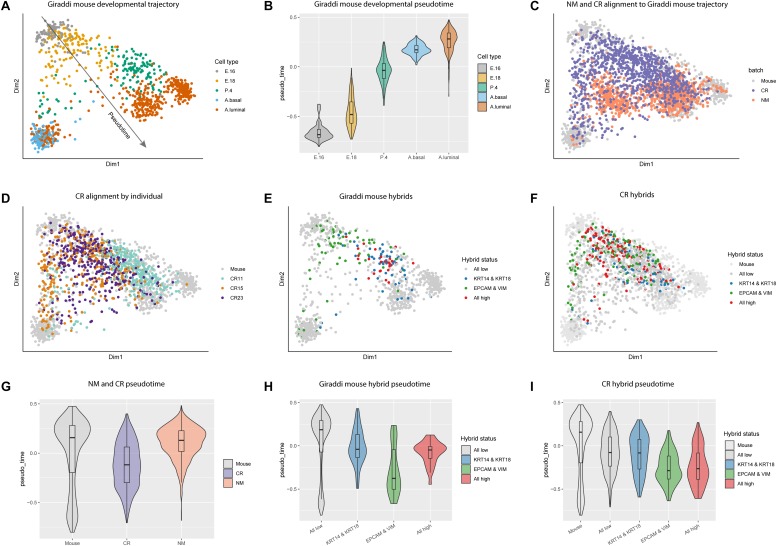
Alignment of NM and CR cells to mouse mammary developmental trajectory and characterization of hybrid cells. **(A)** Principal component analysis plot of single cell RNA-seq data of mouse mammary gland at embryonic day 16 (E.16), embryonic day 18 (E. 18), post-natal day 4 (P.4), and adult basal (A. basal) and adult luminal (A.luminal) cells as reported in Giraddi et al. (2018). **(B)** Pseudotime estimates of mouse mammary developmental stages. **(C)** NM and CR cells mapped to the developmental trajectory with CoRGI. **(D)** CR samples mapped to the mouse mammary developmental trajectory labeled by individual. **(E)** Hybrid cell identification of mouse mammary cells along the developmental trajectory. Luminal/basal hybrids were identified by concurrent high *KRT14/KRT18* expression. Epithelial/mesenchymal hybrids were identified by concurrent high *EPCAM/VIM* expression. Quadruple positive hybrid cells were identified by high expression of all four marker genes *KRT14/KRT18,/EPCAM/VIM.* **(F)** CR cells mapped to mouse developmental trajectory and labeled by hybrid status. **(G)** Pseudotime estimates of NM and CR cells relative to the mouse mammary developmental trajectory cells. **(H)** Pseudotime estimates of mouse hybrid cells. **(I)** Pseudotime estimates of CR hybrid cells.

Due to the enrichment of stem cell associated genes in CR cells, we chose to further investigate this link in the context of mammary gland development. We integrated our data with the mouse mammary single-cell transcriptome atlas generated by Giraddi et al. (2018) which spans mouse mammary gland development from embryonic day 16 to adulthood (Figure 3A; Giraddi et al., 2018). We calculated pseudotime estimates for each cell across the mouse developmental trajectory. Pseudotime estimates correlate to the developmental timepoint during which each cell was isolated, the more negative the pseudotime estimate the more embryonic-like the cell (Figure 3B, Supplementary Table S10). Using the CORGI alignment algorithm, we used the Giraddi data as a reference to map our NM and CR samples onto the mammary gland developmental trajectory. The majority of NM cells aligned to the adult mouse cells, whereas CR cells spanned the trajectory with a distinct population aligning to the embryonic mouse cells (Figure 3C). When CR cells were labeled by individual, CR15 and CR23 had cells spanning the whole trajectory, whereas CR11 mapped mostly to mouse mammary gland at post-natal day 4 and adulthood (Figure 3D).

### Hybrid Stem Cell Populations Emerge Following Conditional Reprogramming

A growing number of studies have characterized hybrid stem cell populations in the normal and cancerous breast and have linked these epithelial/mesenchymal (E/M) or luminal/basal (L/B) hybrid phenotypes to aggressiveness of cancer (Colacino et al., 2018; Gerdur Ísberg et al., 2019; Jolly et al., 2019). Additionally, emerging evidence shows that stem cells can stably exist in hybrid states and that these hybrid phenotypes may be metastable (Jolly et al., 2016). To investigate the presence of hybrid populations in normal mammary cells, we assessed the co-expression of the luminal and basal (here used interchangeably with myoepithelial) markers *KRT18*/*KRT14* (L/B) and the epithelial and mesenchymal markers *EPCAM*/*VIM* (E/M) to identify “double positive” hybrid cells. Overlap of *EPCAM/VIM* and *CDH1/VIM* double positive populations indicate that *EPCAM* and *CDH1* are both effective epithelial markers, however, *EPCAM* was ultimately chosen as the epithelial marker for hybrid identification due to its overall higher expression in NM and CR cells (Supplementary Figures S1G,H). Co-expression of all four markers *KRT18*/*KRT14/EPCAM*/*VIM* identified “quadruple positive” hybrid cells. “Triple positive” hybrid combinations were also assessed, however, we found these redundant and less informative than the double positive and quadruple positive marker combinations (Supplementary Figure S4). We identified L/B, E/M, and quadruple positive hybrid populations in the Giraddi dataset, NM, and CR cells, with CR cells expressing the highest proportions of both double positive hybrids and quadruple hybrids (Supplementary Table S11). CR15 expressed the highest proportion of hybrid cells among the individuals.

In the Giraddi dataset, E/M hybrids spanned both basal and luminal branches of the trajectory, L/B hybrids mostly mapped to adult luminal and post-natal day 4, and quadruple hybrids mapped along the luminal branch around embryonic day 18 and post-natal day 4 (Figure 3E). E/M hybrids were the only cells to map to the basal adult cells and the embryonic cells. To investigate the developmental maturity of hybrid CR cells, we mapped the E/M, L/B, and quadruple hybrids to the mouse developmental trajectory (Figure 3F). Almost all of the hybrid CR cells mapped to mouse cells spanning embryonic day 16 through post-natal day 4, with a few mapping to the adult populations. Interestingly, the hybrid E/M cells map along both the luminal and basal trajectories, however, the L/B hybrids almost exclusively map along the luminal trajectory. The majority of the quadruple hybrids also mapped along the luminal trajectory.

To further characterize the different cell types, pseudotime analysis was performed on the CR, NM, and hybrid populations. Pseudotime estimates for CR cells indicated a more developmentally immature phenotype relative to NM cells (Figure 3G). Pseudotime analysis of the Giraddi mouse hybrid populations revealed that hybrid E/M cells are the most developmentally immature, followed by the quadruple hybrids, and then hybrid L/B cells (Figure 3H). CR hybrids exhibited a similar pattern to the mouse hybrids, where hybrid E/M cells were the most developmentally immature, quadruple hybrids were intermediate, and L/B hybrids were the most mature (Figure 3I). Pseudotime differences between hybrid populations in the CR cells were less pronounced than in the mouse. We also calculated the embryonic stem cell score for the NM and CR hybrid cell populations and found that E/M and quadruple hybrids expressed a higher embryonic stem cell score, whereas L/B hybrids were less distinct (Supplementary Figures S3A–C). From this we concluded that the CR process causes an enrichment of hybrid cells and that these hybrid populations are transcriptionally similar to mammary cells in early development. Finally, E/M and L/B hybrids appear to represent distinct cellular populations with quadruple positive hybrid cells falling somewhere in between.

Differential gene expression analysis between quadruple positive hybrids and all other epithelial NM and CR cells identified 4052 genes upregulated and 2660 genes downregulated in quadruple hybrids (Supplementary Figure S2B and Supplementary Table S12). The most significant DEG upregulated in the quadruple positive hybrids was extracellular matrix gene *COL14A1* which has been found to be upregulated in cancerous breast stroma compared to normal breast stroma (Casey et al., 2009). We further investigated the DEGs from the quadruple hybrids by calculating the overlap of these genes with the MSIGDB EMT hallmark gene set (Supplementary Table S13). We found that 82 out of the 200 (41%) genes differentially expressed in the quadruple hybrids were EMT related genes (Supplementary Figure S2C). Together, these data provide evidence for the presence of hybrid cells in the normal and developing breast, specifically early in development.

### Hybrid Stem Cell Populations Are Enriched During Gestation and Lactation

**FIGURE 4:**
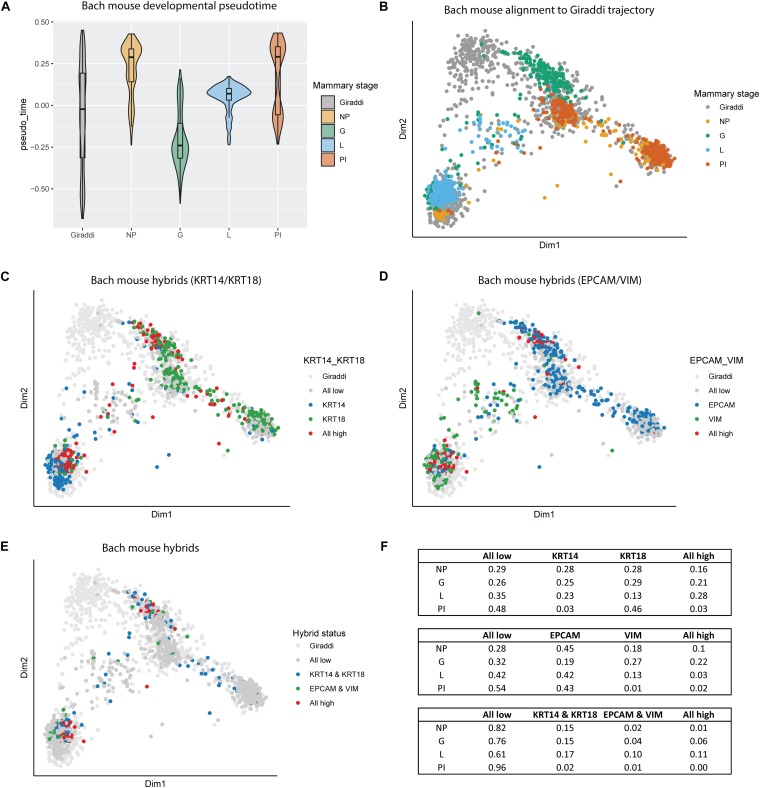
Alignment of Bach mouse mammary developmental dataset to the Giraddi mammary trajectory. **(A)** Pseudotime estimates of Bach mammary developmental stages: nulliparous (NP), mid-gestation (G), lactation (L), and post-involution (PI). **(B)** Bach mammary cells mapped to the Giraddi trajectory with CoRGI. **(C)** Bach luminal/basal hybrid cells mapped to Giraddi trajectory. **(D)** Bach epithelial/mesenchymal hybrid cells mapped to Giraddi trajectory. **(E)** Bach quadruple positive hybrid cells mapped to Giraddi trajectory **(F)** Proportions of Bach hybrid cells by developmental stage.

The enrichment of these hybrid populations early in breast development aligns with the current understanding of the highly dynamic nature of mammary gland morphogenesis. This led us to investigate another highly dynamic and proliferative developmental stage of the breast: gestation and lactation. We incorporated the adult mouse mammary developmental dataset generated by Bach et al. (2017), which spans the nulliparous, mid-gestation, lactation, and post-involution time points (Bach et al., 2017). Alignment of the Bach dataset to the Giraddi developmental trajectory revealed a striking chronological pseudotime arc (Figure 4A). Beginning at the nulliparous stage, mammary cells exhibit a developmentally mature pseudotime, reflected by alignment to Giraddi mouse adult cells. Mammary cells during the gestation stage exhibit a more developmentally immature phenotype, indicated by a decrease in estimated pseudotime. Through the lactation and post-involution stages, pseudotime of mammary cells sequentially increases to stabilize at a pseudotime similar to the developmental maturity of the nulliparous stage. Mapping of these cells to the Giraddi trajectory demonstrated that the nulliparous and post-involution stages mapped most closely to the luminal and basal adult cells, the lactation stage mapped most closely to adult basal cells, and the gestation stage mapped most closely to the embryonic day 18 cells (Figure 4B). L/B, E/M, and quadruple hybrids were also identified in the Bach dataset and mapped to the Giraddi trajectory (Figures 4C–E). Proportions of hybrid cells were calculated for each stage (Figure 4F). The highest proportion of E/M hybrids were found in the gestation stage which also expressed the highest proportion of L/B hybrids, followed by the lactation stage. Interestingly, the lactation stage expressed the highest proportion of quadruple hybrids, followed by the gestation stage. Although the pseudotime estimates for the nulliparous and post-involution stages were similar, the post-involution stage had an approximately 5-fold lower proportion of hybrid cells. The enrichment of hybrid populations during the gestation and lactation stages suggests the importance of these cells during pregnancy-associated mammary gland morphogenesis.

To further extend and validate these findings in human patient samples, we also explored the distribution of hybrid cells in the Nguyen dataset, which is generated largely from nulliparous patients, and compared their alignment to the NM samples (Supplementary Figure 5A). We aligned the Nguyen data to the Giraddi developmental trajectory and found that cells largely clustered with the mouse adult luminal and basal cells (Supplementary Figure S5B). In the Nguyen dataset, there were approximately 11 and 12% of cells classified as E/M and L/B hybrids (Supplementary Figures 5C–J), respectively, which is comparable to the proportion of these hybrid cells in the nulliparous mice from the Bach dataset (10 and 16%). The proportion of these cells in the post-involution mouse cells from the Bach dataset were 3 and 2%, respectively.

### Basal Breast Cancers Are the Most Transcriptionally Distinct and Developmentally Immature of Breast Cancer Subtypes

**FIGURE 5:**
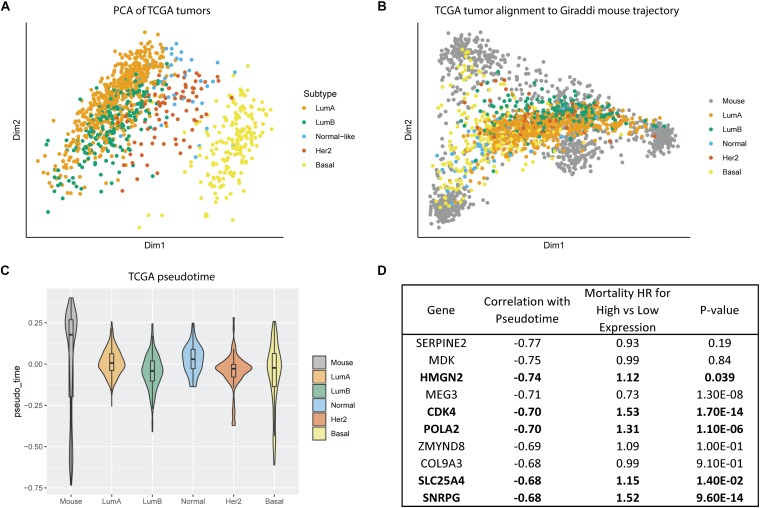
Alignment of TCGA tumors to Giraddi mammary trajectory. **(A)** Principal component analysis of TCGA bulk breast tumor RNA-seq labeled by subtype. **(B)** Alignment of TCGA tumors to Giraddi developmental trajectory. **(C)** Pseudotime estimates of TCGA tumor subtypes. **(D)** Mortality hazard ratio estimates relative to expression of the top 10 genes most negatively correlated with mouse pseudotime. The more negative the pseudotime estimate, the more highly expressed the gene is in the earliest timepoints during development.

All our prior findings about hybrid cell states and developmental phenotypes were characterized in normal human and mouse mammary cells. Our next step was to leverage this data to inform our understanding of breast cancer subtype biology. To do this, we assessed gene expression of breast tumors from the Cancer Genome Atlas (TCGA). Principal component analysis of the TCGA tumors without any alignment showed that basal tumors clustered as the most distinct from the other subtypes, with luminal A and luminal B overlapping, and the other subtypes grouping between the luminal and basal subtypes (Figure 5A). We mapped the bulk TCGA tumor RNA expression data onto the Giraddi mouse developmental trajectory and found that normal, luminal A, and luminal B tumors mapped most closely to the adult cells, HER2 tumors mapped to slightly more immature cells, and basal tumors spanned pseudotime along the basal trajectory (Figure 5B). Pseudotime estimates by subtype revealed that the luminal A subtype exhibits a significantly more developmentally mature phenotype than the luminal B (*p* = 4.97E-07), Her2 (*p* = 0.0495), and basal (*p* < 2E-16) subtypes, with the basal subtype exhibiting the most immature pseudotime estimate (Figure 5C). As a next step, we assessed the link between the pseudotime estimates of gene expression and breast cancer outcomes. Of the top 10 annotated genes with the most negative pseudotime estimates, 5 were significantly associated with poor prognosis in breast cancer patients (Figure 5D). Our results suggest that “phenotypic developmental maturity” of cancer cells, particularly at timepoints strongly associated with the hybrid E/M state may be a distinguishing factor of the subtypes and that pseudotime-associated genes have prognostic implications for breast cancer patients.

## Discussion

Through our integrated analysis of normal human and mouse mammary data and TCGA tumor data, we witness an overarching theme – “developmentally immature” pseudotime is linked to the likelihood of hybrid cells which express a stem-like gene expression signature. We identify an increased proportion of hybrid cells at particular important timepoints during development: in particular the *in utero* period, gestation, and lactation. Others have found associations between an “embryonic stem-cell like” gene expression signature and aggressiveness of cancers (Malta et al., 2018). Hybrid E/M cells present a particularly interesting population to further explore in the context of aggressive cancers due to their low pseudotime estimates and their mapping along the basal mouse trajectory. Together, our results suggest that hybrid cells/states and their stem-like plasticity are important mediators in development and cancer and that this intersection is a promising future direction to explore.

The precision of single-cell RNA-seq allowed us to characterize NM tissue as comprized of stromal, immune, and epithelial cells. When we perturbed NM cells *in vitro* with the conditional reprogramming method, we identified that CR cells only contained luminal and myoepithelial populations, with a small subpopulation of mouse fibroblasts, which were used as a feeder layer to support the growth of the CR cells. The CR process appears to enhance inter-individual heterogeneity, where post-CR samples cluster much more distinctly by individual. Given that the CR samples and NM counterparts were derived from the same individual, the preferential clustering by CR status is indicative that the CR process likely induces major transcriptomic alterations as well as depletion of immune and stromal cells.

DGE analysis between NM and CR samples allowed us to identify a number of significant genes. Understanding their molecular functions may provide crucial mechanistic insight into the CR process, the enrichment of the embryonic stem cell phenotype we observed, and the connection between stemness and cancer. Of these genes, *LGALS1* stands out due to its significant upregulation overall in CR cells as well as in comparisons of DEGs by sample. A member of the galectin family of proteins which modulate proliferation and cell-cell/cell-matrix interactions, upregulation of *LGALS1* expression in breast cancer adjacent fibroblasts has been linked to metastasis and is altered in lymph node metastases compared to primary breast tumors (Feng et al., 2007; Folgueira et al., 2013). Outside of the breast, *LGALS1* is linked to invasiveness and metastasis in oral cancer (Li et al., 2018). Amongst the other highly significant upregulated CR genes by *p*-value and log2FC, *SKA2*, *MKI67*, *HJURP*, *BIRC5*, and *CCNB1* are upregulated in breast cancer tissues and all five except for *SKA2* have been identified as prognostic markers for breast cancer (Li et al., 2013; Ding et al., 2014; Falato et al., 2014; Montes de Oca et al., 2015; Ren et al., 2019; Wang et al., 2019b). Additionally, *BUB1* and *BIRC5* have been linked to stemness, where depletion of *BUB1* reduced cancer stem cell potential in MDA-MB-231 and MCF-7 breast cancer cell lines and *BIRC5* is commonly expressed in embryonic tissues and cancer but not in adult tissues (Han et al., 2015; Ghaffari et al., 2016; Hamy et al., 2016). Experimental evidence continues to support the link between stemness and cancer, and our results showing enrichment of a stem-like phenotype and breast cancer related genes in CR cells adds to this body of work. It is striking that the induction of stem-like proliferation and de-differentiation of normal mammary adult cells by ROCK inhibitor Y-27632 upregulates numerous genes which overlap with breast cancer and metastasis, providing further experimental evidence that cancers are hijacking normal stem cell mechanisms.

Another key finding of our study was the emergence of hybrid cell populations post-CR. Our characterization of these populations is consistent previous reports and provides additional insight into the “developmental maturity” of these hybrid states. Hybrid E/M cells have been found in human primary tumors and lymph nodes where they exhibit enhanced tumor initiation and metastatic potential and are implicated in therapy resistance and poor survival (Yu et al., 2013; Grosse-Wilde et al., 2015; Jolly et al., 2019). Similarly, L/B hybrids have been characterized in both normal and cancer tissue from humans and are believed to be derived from luminal progenitors (Abd El-Rehim et al., 2004; Gerdur Ísberg et al., 2019). This hypothesis of L/B hybrids being luminal in origin is consistent with our observations, where L/B hybrids in both human and mouse map only along the luminal trajectory of the mouse mammary gland developmental atlas, whereas E/M hybrids map to both luminal and basal trajectories. Sun et al. (2010) have shown that in the developing mouse mammary gland, *KRT5*/*KRT14* (L/B) hybrids are observed beginning from embryonic day 15.5 up until adulthood (8–12 weeks). While these populations decreased after 3 weeks, it is important to note that they were still present in the normal adult mouse mammary gland. Additionally, Sun et al. (2010) also identified a distinct population of cells expressing *KRT6*, a multipotent mammary epithelial progenitor marker, which emerged at embryonic day 16.5 and was localized to the nipple sheath. Expression of *KRT6* was also correlated to the boundary of the mammary mesenchyme, separate from luminal and basal localization. Considering the proximity to the mammary mesenchyme and its distinctness from luminal and basal progenitors, the *KRT6* population in the mouse mammary gland may be analogous to the E/M hybrids we identified in the CR population. The embryonic origin of hybrid populations in the developing mouse mammary gland and their persistence through adulthood suggests that hybrid populations in the human mammary gland also arise during embryogenesis and are maintained through adulthood.

Pseuodotime analysis of mouse, NM, CR, and breast tumor samples suggest that the “developmental maturity” state of a cell or tumor plays a direct role in its biological behavior. Of the hybrid populations in both mouse and CR cells, the E/M hybrids exhibited the lowest estimated pseudotime. Based on prior knowledge implicating E/M hybrids in tumorigenesis and metastasis, this population may be of particular interest in the future to target for cancer prevention and therapy. To understand the impact of variation in pseudotime on our understanding of breast subtype biology, we calculated pseudotime estimates of bulk tumor RNA-seq data from TCGA samples. On average, none of the TCGA tumor subtypes exhibited pseudotime scores corresponding to adult mouse cells. Instead, average subtype scores corresponded to post-natal day 4 and earlier in development. While these are bulk samples being aligned to single-cell mouse samples, this suggests that regardless of cell type, a more developmentally immature phenotype is characteristic of cancers. Among the subtypes, basal cancers preferentially map to the most developmentally immature cells in the mammary gland and express the lowest pseudotime scores. This difference in “developmental maturity” may be a key distinction between basal cancers and other subtypes and may play a major role in the aggressiveness and low survival outcomes observed clinically and epidemiologically.

One of our most exciting findings was the characterization of hybrid cells in the adult mouse mammary gland during pregnancy. The enrichment of hybrid populations during gestation and lactation and their loss in the subsequent post-involution stage suggests that these hybrid states are inducible and transient. This transiency provides compelling evidence that these hybrid populations are instrumental to the dynamic modifications in breast morphogenesis which occur during pregnancy and lactation. This arc of mouse hybrid enrichment and stabilization parallels the transient increase in breast cancer risk during and immediately following pregnancy, which decreases over time. The time period during which hybrid populations are most prevalent in the mouse breast overlaps with pregnancy associated breast cancer (PABC) risk in humans, diagnosed between pregnancy and 1 year following birth (Ruiz et al., 2017). This overlap in time period, as well as the parallel transiency of mouse hybrid populations and PABC risk, supports the presence of these hybrid populations in the human breast during pregnancy and implicates their involvement in PABC. The pathophysiology of PABC is characterized by metastatic, high grade tumors, and survival is inversely correlated with time since birth (Ruiz et al., 2017). Consistent with this is the finding that ER-/PR-/HER2+, and triple-negative tumors are more common in women diagnosed with PABC compared to nulliparous women (Johansson et al., 2018). Based on our other findings that basal breast tumors exhibit the most “developmentally immature” pseudotime estimates and the link between hybrid cells and aggressive cancers, characterizing hybrid populations and “developmental maturity” of PABCs could inform prognostic and therapeutic treatment.

Our study had a number of limitations. One was the source and sample size of mammary tissue. Mammoplasty tissue has been critiqued as not being fully representative of the “normal” breast. Due to the de-identification of the samples we also lack demographic data on the women from whom they were obtained for our study, although we were able to supplement our findings with additional human data from the Nguyen study (Degnim et al., 2012). Moreover, the conditional reprogramming methodology only supports the outgrowth of epithelial cells from samples, a phenomenon which has been linked to the J2 fibroblast co-cultures since the 1970s (Rheinwatd and Green, 1975). A better understanding of stromal/epithelial interactions in regulating these hybrid stem cell states is an important future direction of research. These future experiments could, for example, assess the impact of adult fibroblasts or cancer associated fibroblasts on the reprogramming process. Future complementary analyses of conditional reprogramming using breast cancer samples could also provide important insights into the impacts of enhanced stemness and developmental immaturity on tumor characteristics. While single-cell technology is rapidly evolving and improving, we acknowledge that in this study we are only capturing expression of a subset of the genes expressed in each individual cell. Another limitation is the potential for unanticipated bias from using the subset of CORGI selected genes for alignment with the human mammary cells and TCGA tumor samples to the mouse developmental trajectory.

Overall, we showcase a computational analysis which leverages publicly available data to gain insight into the relationship between hybrid cell populations, stemness, and cancer. We and others have identified significant inter-individual heterogeneity in proportions of stem cells in mammary tissue (Nakshatri et al., 2015; Colacino et al., 2018). Our ongoing work is utilizing single-cell RNA-seq of normal mammary tissue from epidemiologically well characterized women to understand how known epidemiological risk factors for cancer influence the “stemness” of breast epithelial cells. Quantification of reprogramming efficiency during conditional reprogramming across samples from diverse women could provide a functional readout of “stemness” or reprogramming capacity and their relations to known cancer risk factors, such as age, ethnicity, or genetic predisposition to cancer. Future work can focus on identifying the localization of these hybrid states in the adult mammary gland using advanced techniques, such as spatial transcriptomics. Overall, these results provide further evidence to support investigating the role of stem cells, and particularly hybrid E/M cells, in normal development and characterizing how this biology is hijacked during tumorigenesis. Understanding the biology of these cells will likely provide novel targets for the prevention and therapy of aggressive breast cancers.

## Data Availability Statement

The datasets generated for this study can be found in the Gene Expression Omnibus (GSE146792).

## Ethics Statement

The studies involving human participants were reviewed and approved by the study protocol was reviewed and approved by the University of Michigan Institutional Review Board (HUM00042409). Written informed consent for participation was not required for this study in accordance with the national legislation and the institutional requirements.

## Author Contributions

TT conducted the experiments, analyzed the data, and wrote the manuscript. YW developed the analysis methods, analyzed the data, and wrote the manuscript. MB conducted the experiments, analyzed the data, and wrote the manuscript. CL analyzed the data. CS developed the analysis methods. LB developed the analysis methods. MW conceptualized the study and interpreted the data. JC conceptualized the study, analyzed the data, and wrote the manuscript. All authors reviewed and approved the final version of the manuscript.

## Conflict of Interest

The authors declare that the research was conducted in the absence of any commercial or financial relationships that could be construed as a potential conflict of interest.

## Supplementary Material

FIGURE S1 Post-CR single cell unbiased clustering and gene expression. **(A)** tSNE dimension reduction of CR cells colored by cell cluster, identified by unbiased clustering **(B)** Expression of known cell type marker genes by cluster. Cluster 7 and 8 identified as mouse cells **(C)** tSNE dimension reduction of NM and CR samples by individual. Each individual sample was down-sampled to 200 cells. **(D)** NM and CR FeaturePlots of myoepithelial marker gene (*KRT14*) and **(E)** luminal marker gene (*KRT18*) expression **(F)** Expression of known cell type marker genes by down-sampled NM and CR individuals **(G)** Identification of NM and CR *CDH1/VIM* double positive cells and **(H)** *EPCAM/VIM* double positive cells. Table compares the overlap between the *CDH1/VIM* and *EPCAM/VIM* classifications.

FIGURE S2 Post-CR differential gene expression and pathway analysis. **(A)** Comparison of differentially expressed genes between NM and CR cells of individual samples. DEGs are plotted by average log2FC. Positive values represent genes upregulated in CR and negative values represent genes downregulated in CR. **(B)** Differential gene expression of quadruple hybrids vs. all other NM and CR cells. Significantly upregulated genes in quadruple positive hybrids (FDR < 0.05) are colored in orange. Significantly downregulated genes in quadruple hybrids are colored in purple. **(C)** Overlap between quadruple hybrid upregulated genes and EMT related genes. Upregulated EMT genes in quadruple hybrids in orange, and downregulated EMT genes in purple. **(D)** Comparison of overlap between NM and CR differentially expressed genes and the luminal progenitor gene expression signature reported in Lim et al. (2009). Yellow genes indicate luminal progenitor genes more highly expressed in CR vs. NM. **(E)** Comparison of overlap between NM and CR differentially expressed genes and the ROCK pathway gene set. **(F)** Top 10 transcription factors associated with top 1000 genes overexpressed in CR cells.

FIGURE S3 Embryonic stem cell gene signature of NM and CR hybrid cells. **(A)** ESC score of NM and CR cells labeled by *EPCAM/VIM* hybrids status, **(B)** *KRT14/KRT18* hybrid status, and **(C)** quadruple positive hybrid status.

FIGURE S4 Comparison of triple positive Giraddi mammary cells and aligned CR cells. Localization of **(A)** mouse *KRT14/EPCAM/VIM* triple positive cells, **(B)** CR *KRT14/EPCAM/VIM* triple positive cells, **(C)** mouse *KRT14/KRT18/EPCAM* triple positive cells, **(D)** CR *KRT14/KRT18/EPCAM* triple positive cells, **(E)** mouse *KRT14/KRT18/VIM* triple positive cells, **(F)** CR *KRT14/KRT18/VIM* triple positive cells, **(G)** mouse *KRT14/EPCAM/VIM* triple positive cells, and **(H)** CR *KRT14/EPCAM/VIM* triple positive cells when aligned to the mouse mammary developmental trajectory with CoRGI.

FIGURE S5 Comparison of hybrid NM cells and Nguyen human mammary cells. **(A)** Alignment of NM cells to Giraddi trajectory by individual. **(B)** Alignment of Nguyen mammary cells to Giraddi trajectory. **(C)** NM *EPCAM/VIM* hybrids. **(D)** Nguyen *EPCAM/VIM* hybrids. **(E)** NM KRT14/KRT18 hybrids **(F)** Nguyen KRT14/KRT18 hybrids. **(G)** NM quadruple positive hybrids. **(H)** Nguyen quadruple positive hybrids **(I)** Proportions of NM cells by hybrid status. **(J)** Proportions of Nguyen cells by hybrid status.

TABLE S1 NM cluster markers. Most highly expressed genes for each individual NM cluster, orderd by log2FC.

TABLE S2 CR cluster markers. Most highly expressed genes for each individual CR cluster, orderd by log2FC.

TABLE S3 CR vs. NM differential gene expression. Differentially expressed genes between pooled epithelial NM and CR cells. Genes are ordered by log2FC, with higher numbers corresponding to genes up in CR and vice versa.

TABLE S4 CR vs. NM differential gene expression by individual. Differentially expressed genes between the NM and CR cells of each individual ordered by log2FC.

TABLE S5 ROCK gene set. Rho-associated kinase genes used to characterize ROCK enrichment between NM and CR.

TABLE S6 CR transcription factors. ENCODE and CHEA transcription factors identified from the top 1000 differentially expressed genes in CR.

TABLE S7 ESC gene set. List of genes from the Gene Set Enrichment Analysis used to calculated an embryonic stem cell gene expression score for NM and CR cells.

TABLE S8 Mammary stem cell gene set. List of mammary stem cell genes used to characterize mammary stem cell enrichment between NM and CR.

TABLE S9 Luminal progenitor gene set. List of luminal progenitor genes used to characterize luminal progenitor enrichment between NM and CR.

TABLE S10 Mouse pseudotime genes. Arrow-of-time vector genes which most differentiate embryonic mouse mammary cells and adult cells from the Giraddi dataset. The lower the pseudotime score the more embryonically associated the gene and vice versa.

TABLE S11 Hybrid proportions. Proportion of hybrid cells in Giraddi mouse dataset and NM and CR.

TABLE S12 Quadruple hybrids vs. all other epithelial differential gene expression. Differentially expressed genes between quadruple hybrids and all other epithelial cells.

TABLE S13 Overlap between differentially expressed genes in quadruple hybrids and EMT genes. A subset of differentially expressed genes between quadruple hybrids and all other epithelial cells which have been identified as EMT genes.

TABLE S14 Mouse pseudotime vs. CR expression. Arrow-of-time mouse pseudotime genes applied to genes expressed in CR cells.
