# Supplementary figures and images for: Hybrid Stem Cell States: Insights Into the Relationship Between Mammary Development and Breast Cancer Using Single-Cell Transcriptomics
Source: Front Cell Dev Biol. 2020 May 8;8:288. doi: 10.3389/fcell.2020.00288 (PMC7227401; doi:10.3389/fcell.2020.00288)

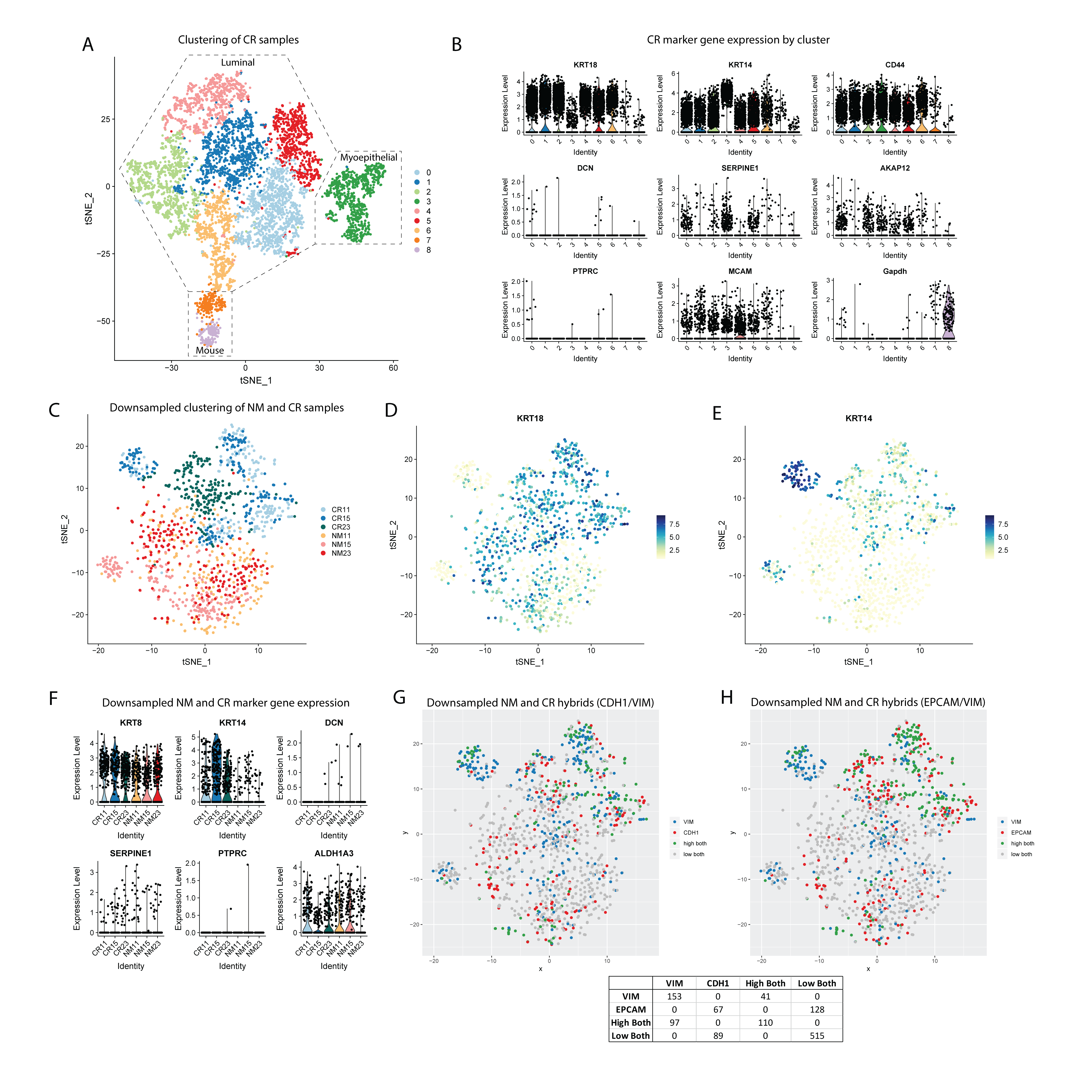

Supplement: FIGURE S1 — Post-CR single cell unbiased clustering and gene expression. (A) tSNE dimension reduction of CR cells colored by cell cluster, identified by unbiased clustering (B) Expression of known cell type marker genes by cluster. Cluster 7 and 8 identified as mouse cells (C) tSNE dimension reduction of NM and CR samples by individual. Each individual sample was down-sampled to 200 cells. (D) NM and CR FeaturePlots of myoepithelial marker gene (KRT14) and (E) luminal marker gene (KRT18) expression (F) Expression of known cell type marker genes by down-sampled NM and CR individuals (G) Identification of NM and CR CDH1/VIM double positive cells and (H) EPCAM/VIM double positive cells. Table compares the overlap between the CDH1/VIM and EPCAM/VIM classifications. [file Image_1.TIF]

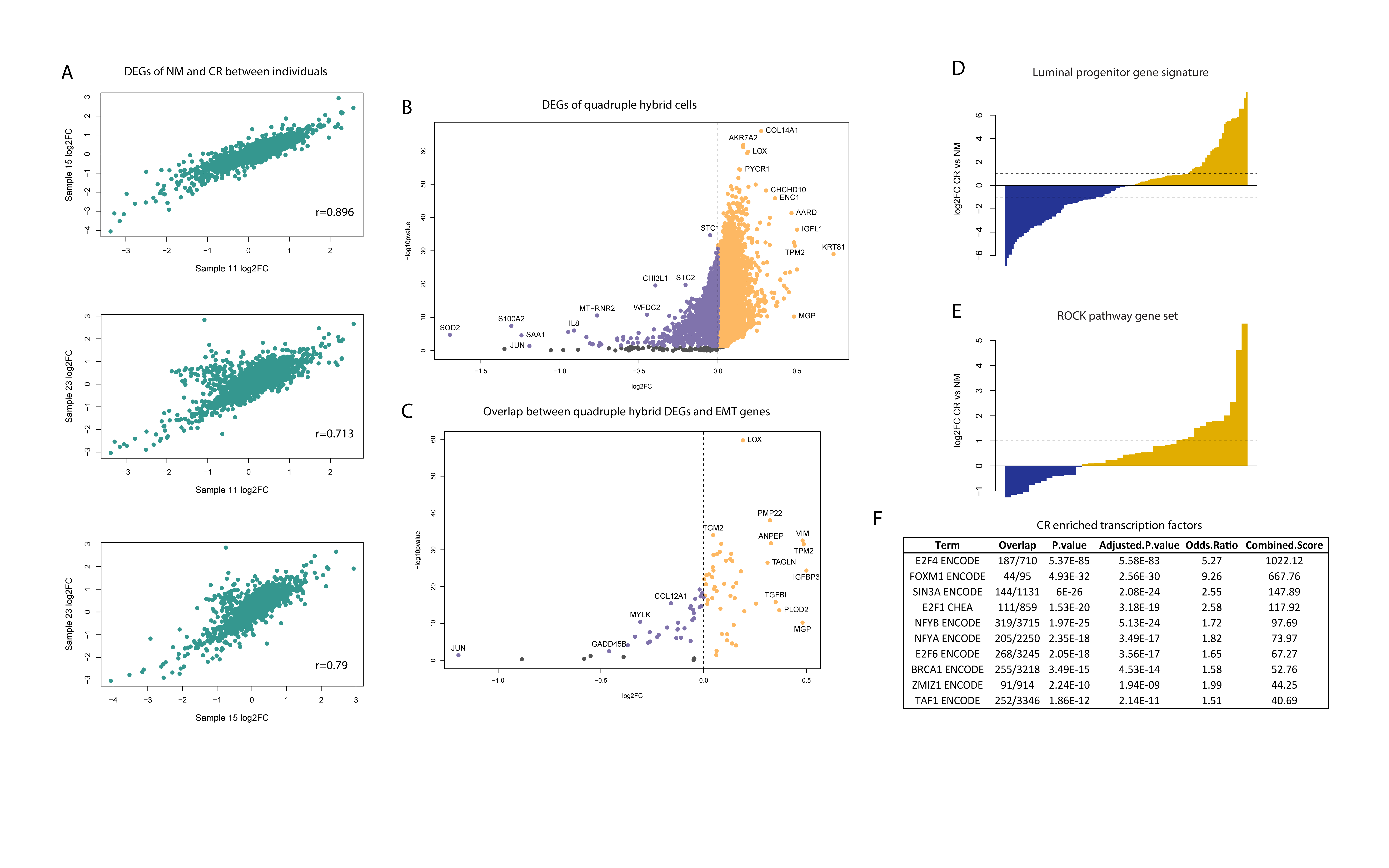

Supplement: FIGURE S2 — Post-CR differential gene expression and pathway analysis. (A) Comparison of differentially expressed genes between NM and CR cells of individual samples. DEGs are plotted by average log2FC. Positive values represent genes upregulated in CR and negative values represent genes downregulated in CR. (B) Differential gene expression of quadruple hybrids vs. all other NM and CR cells. Significantly upregulated genes in quadruple positive hybrids (FDR < 0.05) are colored in orange. Significantly downregulated genes in quadruple hybrids are colored in purple. (C) Overlap between quadruple hybrid upregulated genes and EMT related genes. Upregulated EMT genes in quadruple hybrids in orange, and downregulated EMT genes in purple. (D) Comparison of overlap between NM and CR differentially expressed genes and the luminal progenitor gene expression signature reported in Lim et al. (2009). Yellow genes indicate luminal progenitor genes more highly expressed in CR vs. NM. (E) Comparison of overlap between NM and CR differentially expressed genes and the ROCK pathway gene set. (F) Top 10 transcription factors associated with top 1000 genes overexpressed in CR cells. [file Image_2.TIF]

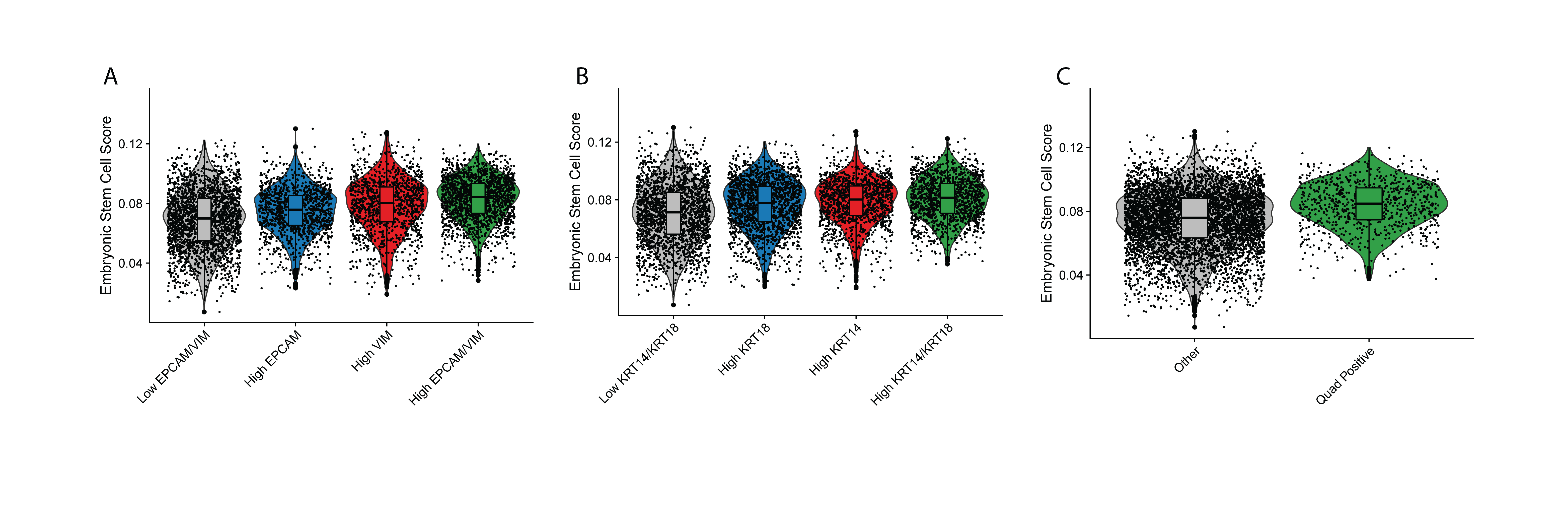

Supplement: FIGURE S3 — Embryonic stem cell gene signature of NM and CR hybrid cells. (A) ESC score of NM and CR cells labeled by EPCAM/VIM hybrids status, (B) KRT14/KRT18 hybrid status, and (C) quadruple positive hybrid status. [file Image_3.TIF]

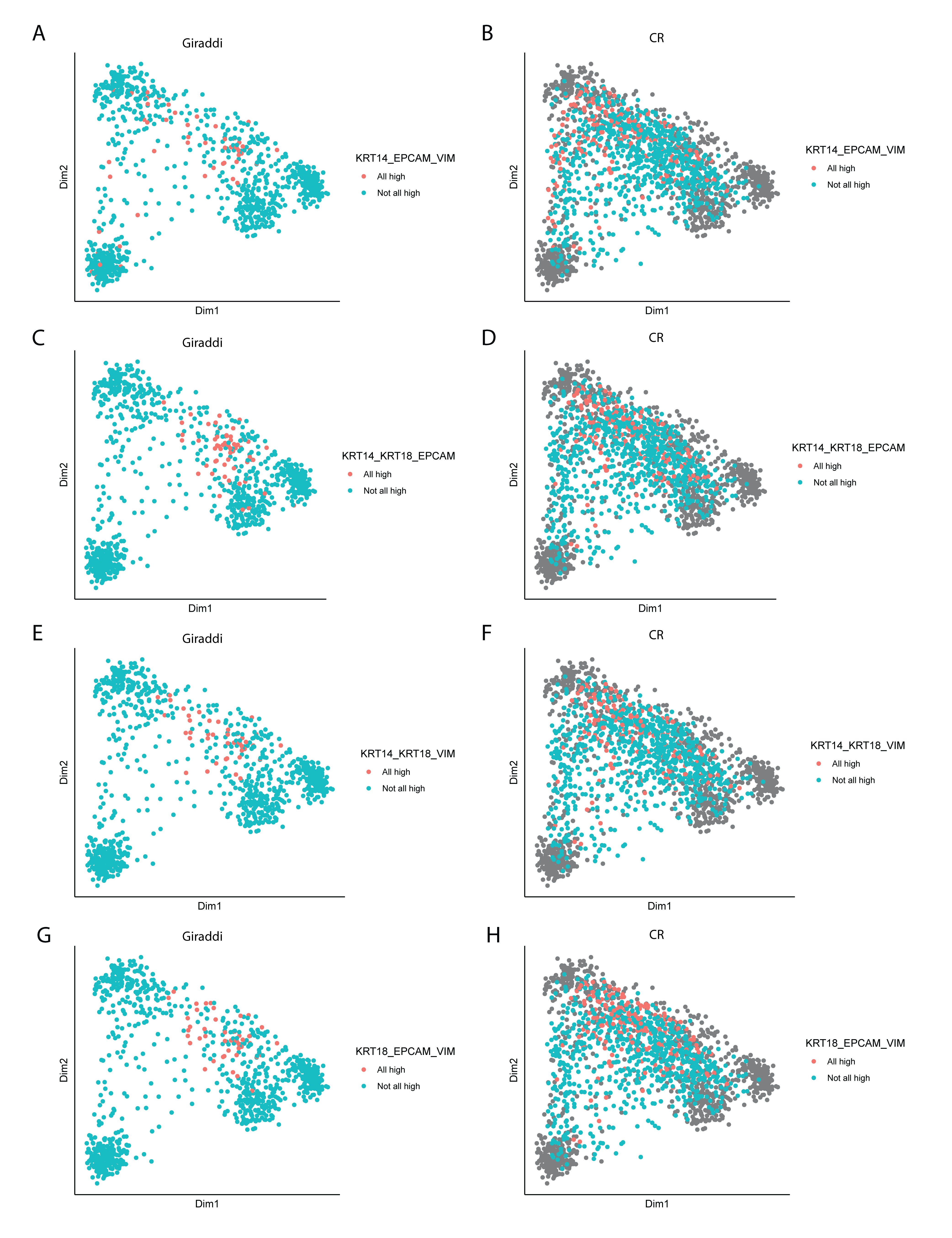

Supplement: FIGURE S4 — Comparison of triple positive Giraddi mammary cells and aligned CR cells. Localization of (A) mouse KRT14/EPCAM/VIM triple positive cells, (B) CR KRT14/EPCAM/VIM triple positive cells, (C) mouse KRT14/KRT18/EPCAM triple positive cells, (D) CR KRT14/KRT18/EPCAM triple positive cells, (E) mouse KRT14/KRT18/VIM triple positive cells, (F) CR KRT14/KRT18/VIM triple positive cells, (G) mouse KRT14/EPCAM/VIM triple positive cells, and (H) CR KRT14/EPCAM/VIM triple positive cells when aligned to the mouse mammary developmental trajectory with CoRGI. [file Image_4.TIF]

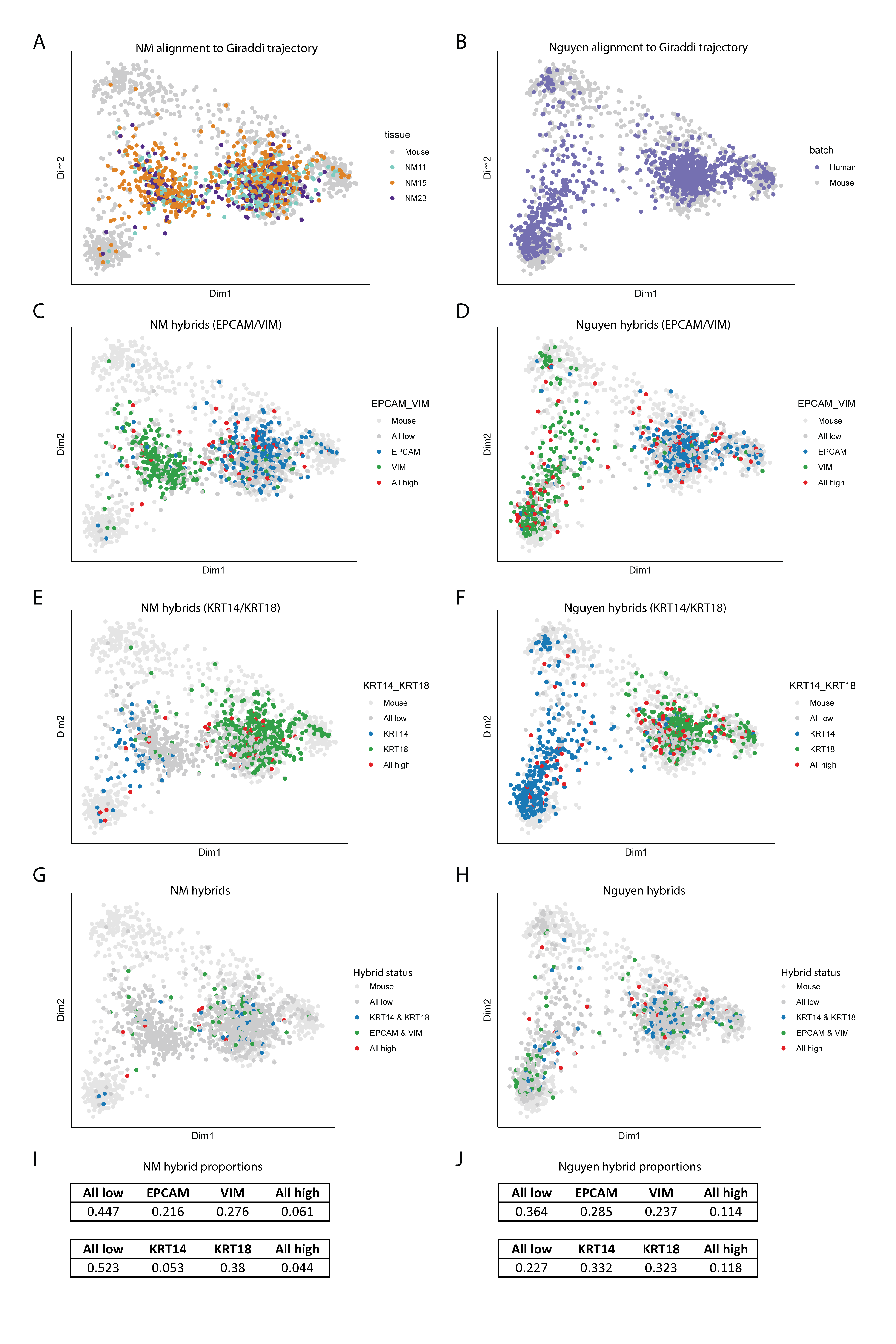

Supplement: FIGURE S5 — Comparison of hybrid NM cells and Nguyen human mammary cells. (A) Alignment of NM cells to Giraddi trajectory by individual. (B) Alignment of Nguyen mammary cells to Giraddi trajectory. (C) NM EPCAM/VIM hybrids. (D) Nguyen EPCAM/VIM hybrids. (E) NM KRT14/KRT18 hybrids (F) Nguyen KRT14/KRT18 hybrids. (G) NM quadruple positive hybrids. (H) Nguyen quadruple positive hybrids (I) Proportions of NM cells by hybrid status. (J) Proportions of Nguyen cells by hybrid status. [file Image_5.TIF]
